# Evaluation of preprocedural statin loading on clinical outcomes in patients undergoing elective percutaneous coronary intervention

**DOI:** 10.3389/fcvm.2024.1435989

**Published:** 2024-08-20

**Authors:** Umit Yasar Sinan, Bengisu Keskin Meric, Nurbanu Bursa, Gkiozde Moumin, Aysem Kaya, Alev Arat Ozkan

**Affiliations:** ^1^ Department of Cardiology, Istanbul University-Cerrahpasa Institute of Cardiology, Istanbul, Türkiye; ^2^ Department of Statistics, Faculty of Science, Hacettepe University, Ankara, Türkiye; ^3^ Department of Biochemistry, Istanbul University-Cerrahpasa Institute of Cardiology, Istanbul, Türkiye

**Keywords:** myocardial necrosis, elective coronary angioplasty, rosuvastatin, Peri-procedural Myocardial Infarction, prevention

## Abstract

**Background and aim:**

High-dose statin therapy before percutaneous coronary intervention (PCI) is thought to reduce the occurrence of Peri-procedural Myocardial Infarction (PPMI), which is associated with increased mortality and prolonged hospitalization, especially in statin naïve patients. This study aims to investigate the effect of rosuvastatin loading dose on PPMI and major adverse cardiac and cerebrovascular events (MACCE) in patients undergoing elective PCI, considering their statin use.

**Methods:**

One hundred sixty-five patients with stable coronary artery disease (CAD) without heart failure (HF) or chronic kidney disease (CKD) were included in the study. They were divided into two groups: patients already on statin treatment (*n*:126) and statin naive patients (*n*:39). Both groups were randomly assigned to high-dose (40 mg) rosuvastatin (*n*:86) or a non- loading dose group (*n*:79). The primary endpoint was the incidence of PPMI, and the secondary endpoint was MACCE.

**Results:**

The mean age of study population was 59 ± 9.4 years with 77% being male (*n* = 127). The median follow-up (FU) time was 368 day. Thirty patients were diagnosed with PPMI after PCI (19 in the high-dose group and 11 in the no-loading-dose group). Meanwhile, less than half of study population (77 patients, 46.7%) had complex lesion type (B2, C) and 88 of those (53.3%) had simple lesion type (A, B1). PPMI was observed more frequently in statin-naive patients (23%) than in statin users (17%), although the difference was not statistically significant. Only two patients (1.2%) experienced MACCE during the FU period. One of these patients, who had a type C lesion, belonged to group A2 and underwent Target Vessel Revascularization (TVR) on the 391st day. The other patient, with a type B1 lesion, was in group A1 and was hospitalized due to Acute Coronary Syndrome (ACS) on the 40th day of FU.

**Conclusions:**

Pre-procedural administration of high dose rosuvastatin in patients with stable coronary artery disease did not decrease PPMI, independent of chronic statin use.

## Introduction

Peri-Procedural Myocardial Infarction (PPMI), occurring in approximately 15%–20% of patients undergoing Percutaneous Coronary Intervention (PCI), stands as a significant complication with far-reaching consequences. Its pathophysiology involves the distal embolization of fragile coronary plaque fragments, leading to heightened short- and long-term mortality rates attributed to major adverse cardiac and cerebrovascular events (MACCE) (1). The predictors of PPMI include advanced age, multi-vessel disease (MVD), diabetes mellitus (DM), and the complexity of coronary artery disease (CAD) (2–8).

Beyond their renowned lipid-lowering effects, statins have pleiotropic effects, including the enhancement of endothelial function, mitigation of oxidative stress and inflammation, and vasodilation of coronary microvascular flow (9, 10). Loading doses of statins, particularly rosuvastatin, have emerged as a potential intervention to mitigate PPMI, as underscored by trials like Secure-PCI and the ROMA Trial (1, 11). However, despite promising evidence, further investigation is warranted to validate the hypothesis surrounding the purported superior pleiotropic effects of rosuvastatin compared to other statins.

The primary aim of our study was to assess the impact of a high loading dose of rosuvastatin on the incidence of PPMI and subsequent MACCE in a specific cohort from a tertiary university hospital: patients diagnosed with stable CAD, without heart failure (HF) and chronic kidney disease (CKD), undergoing elective PCI, with and without a history of chronic statin use. As a secondary, exploratory analysis, we also aimed to identify characteristics associated with PPMI. This exploratory analysis was not pre-defined and was conducted to generate hypotheses for future research.

## Material and methods

**Figure 1:**
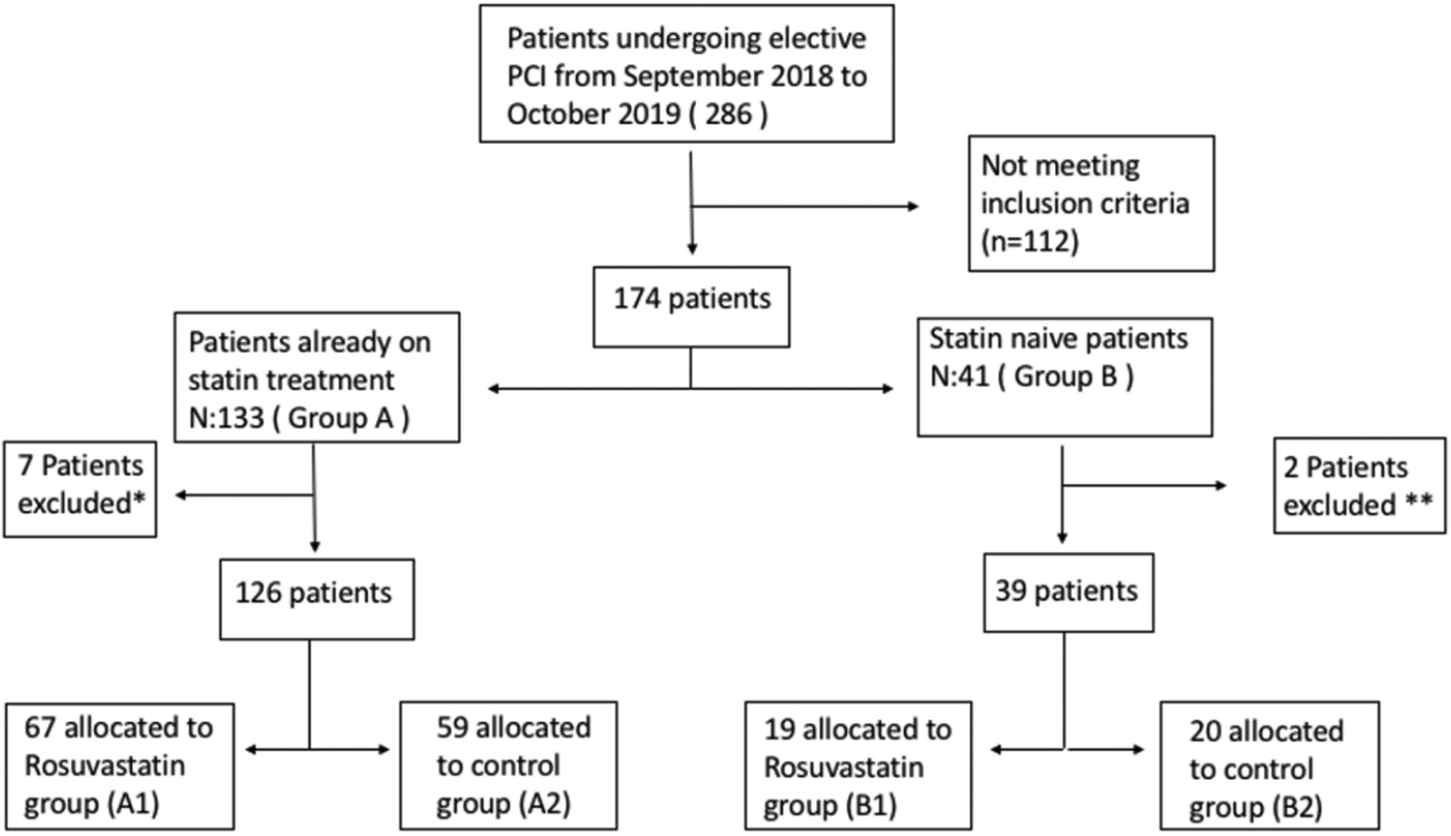
Flow chart of the study. *: 5 had coronary angiography alone, 2 were referred to CABG. **: 1 had coronary angiography alone, 1 were referred to CABG.

From a pool of 826 patients who underwent PCI between September 2018 and October 2019 in Istanbul University-Cerrahpasa Institute of cardiology, a tertiary university hospital in Istanbul, Turkey, 286 were screened in this single-center, prospective trial. Exclusion criteria included acute coronary syndrome (ACS), elevated basal troponin levels, CKD (patients with glomerular filtration rate < 60 ml/minute/m^2^), left ventricular ejection fraction (LVEF) < 40%, congenital heart disease (CHD), severe native or prosthetic valve disease, coronary artery by-pass grafting (CABG) requirement and a history of cancer. After exclusion of these patients, 165 patients finally were categorized into two groups: those already on statin treatment (statin prevalent) (Group A) and statin-naive patients (those who have not used statin in the last year) (Group B). Both groups consecutively were randomized to receive either a high-dose (40 mg) loading dose of rosuvastatin (Groups A1, B1) or no loading dose (Groups A2, B2) and were included in this study (Figure 1). In group B2 (statin naïve patients without loading dose), patients were initiated rosuvastatin without loading dose according to their baseline LDL-C levels, as they have clear evidence of CAD. There was no permutation block for randomization. High dose statin were given a day before PCI procedure.

The primary objective of this study is to assess the impact of a loading dose of rosuvastatin on the incidence of Peri-Procedural Myocardial Infarction (PPMI) in patients undergoing elective Percutaneous Coronary Intervention (PCI). Additionally, the study aims to evaluate the demographics and clinical characteristics of patients who experience PPMI events. The secondary endpoint is the occurrence of subsequent Major Adverse Cardiac and Cerebrovascular Events (MACCE). According to the “Fourth Universal Definition of Myocardial Infarction” guideline of the European Society of Cardiology (ESC), PPMI is defined as a five-fold increase in cardiac troponin (cTn) values in patients with normal baseline troponin levels, or a 20% increase in patients with elevated baseline levels, within 48 h after PCI. Additional criteria for diagnosis include objective indicators of myocardial ischemia, such as ischemic symptoms lasting at least 20 min, new ST segment changes, new pathological Q waves, new left bundle branch block, loss of coronary patency, presence of no reflow, distal embolization, slow flow on angiography, or new onset wall motion abnormalities (12). The MACCE comprises all-cause mortality, stroke or cerebrovascular accident, nonfatal myocardial infarction, target vessel revascularization (TVR), and acute coronary syndrome. TVR refers to revascularization procedures involving the primary treated artery, while stroke is defined as a permanent neurologic deficit confirmed by a neurologist and confirmed by magnetic resonance imaging (MRI). Spontaneous myocardial infarction is characterized by ischemia resulting from a primary coronary event, such as plaque erosion or rupture.

Percutaneous coronary intervention procedures and stent implantations were performed according to the recommendations from the Society of Coronary Angiography and Intervention (SCAI). An expert interventional cardiologist within the study team evaluated coronary angiography and PCI procedures. Lesion characteristics, including location, number, percentage of stenosis, and presence of thrombus, were documented. Lesion complexity was classified according to the American Heart Association (AHA) Classification (13). Angiographic success was defined as final residual restenosis <20% and Thrombolysis In Myocardial Infarction (TIMI) III flow grade. Procedural complications, such as coronary perforation, side branch occlusions, coronary artery dissection, and slow or no-reflow phenomenon, were recorded. Drug-eluting stents (DES) were universally implanted. Baseline high-sensitivity Troponin T (hs-TnT) and electrocardiogram (ECG) measurements were obtained at admission, with subsequent blood samples collected at 12- and 24 h post-PCI intervals. Preprocedural total and LDL cholesterol levels were assessed, and statin therapy was adjusted according to ESC guidelines based on LDL levels. Other treatments, including anti-ischemic and antihypertensive therapies, were managed by physicians according to institutional protocols and guidelines.

Venous blood samples were collected before and after PCI at 12- and 24 h intervals to measure hs-TnT levels using the Cobas E411 hormone analyzer (Roche Diagnostics GmbH, Germany) with the 4th generation Elecsys Troponin T kit.

Patient data during hospitalization were extracted from medical records, with planned follow-up visits scheduled at 15 days, 6 months, and 12 months post-procedure to evaluate MACCE. For patients who missed scheduled hospital visits, E-pulse records evaluation and telephone visits were conducted to inquire about outcomes.

Based on the literature cited, we expected PPMI events in approximately 20% of our patient population; even detecting a 50% reduction in the prevalence of PPMI (down to 10%) with 80% power would require approximately 200 patients in each treatment group. However, our study enrolled 165 patients total. The COVID-19 pandemic and other logistical challenges affected patient enrollment and study duration.

The study was approved by a local ethics committee (Istanbul University-Cerrahpasa Ethic Committee, 38082516-900-79311 approval code and 22.10.2018 approval date) and every observed patient gave written informed consent.

## Statistical analysis

Statistical analyses were conducted using SPSS 23 for Windows (SPSS Inc., Chicago, IL, USA). Whether the distribution of a characteristic follows a normal distribution was evaluated using the Kolmogorov-Smirnov (for larger sample size) or Shapiro-Wilk test (for smaller sample size). Continuous variables, according to having normal distribution or not, were demonstrated as mean ± standard deviation or median (quartile deviation), respectively. Categorical variables were demonstrated as percentages. In our study, we conducted both unadjusted and adjusted analyses to explore potential associations between various patient characteristics and PPMI incidence. Univariate analyses were performed using chi-square tests for categorical variables and *t*-tests for continuous variables. Multivariate logistic regression was used to identify independent predictors of PPMI, adjusting for potential confounders such as age, gender, diabetes mellitus, hypertension, and lesion complexity.

For the multivariate logistic regression model, only baseline (pre-procedural) variables with a *p*-value < 0.1 in univariate analyses were considered for entry into the model. Post-procedural variables, such as post-PCI hs-TnT levels, were not included in the model, as these measures are outcomes and not predictors. This approach ensures that the model only includes variables that are available before the PCI procedure and can be used to predict PPMI. Baseline hs-TnT levels were allowed to enter the model if they met the *p*-value threshold.

Comparisons between patients already on statin treatment and statin-naive patients were conducted to explore potential differences in outcomes. It is important to note that these comparisons are inherently subject to confounding due to the non-randomized nature of previous statin use. To address potential confounding, we conducted both unadjusted and adjusted analyses. The unadjusted analyses provided an initial overview of the associations, while the adjusted analyses accounted for confounding variables such as age, gender, diabetes mellitus, hypertension, and lesion complexity. Despite these adjustments, residual confounding may still be present, and the results should be interpreted with caution. The primary endpoint of the study was the incidence of PPMI, and the secondary endpoint was MACCE. Statistical analyses were performed using SPSS version 23.0, and significance was set at *p* < 0.05.

In addition to the primary analysis, we conducted an exploratory analysis to identify characteristics associated with PPMI. Given the exploratory nature of this analysis, it was not pre-defined in our study protocol. We performed multiple comparisons to investigate various potential predictors of PPMI. However, we acknowledge that multiple comparisons increase the risk of Type I error. To account for this, we considered the exploratory findings as hypothesis-generating and recommend cautious interpretation. We did not apply formal adjustments for multiple comparisons, such as the Bonferroni correction, given the exploratory nature of this analysis.

## Results

Patients that were scheduled for elective coronary angiography/angioplasty (*N* = 286) were screened. One hundred twenty one patients who did not fulfill the inclusion criteria, had only angiography or referred to CABG were excluded. After exclusion, the patients were classified according to their statin use and then they were randomized into patients receiving rosuvastatin loading dose or patients receiving no rosuvastatin loading dose. The flowchart of the study is shown in Figure 1.

The mean age of study population was 59 ± 9.4 with 77% being male (*n* = 127). Median follow-up time was 368 days. There were 60 patients (36.4%) with DM, 84 patients (51%) with HT and two thirds of study population (64%) have previous history of CAD. While 77 patients (46.7%) had a complex lesion (B2, C), 88 patients (53.3%) had a simple lesion (A, B1). A total of 67 patients (40.6%) were classified to A1, 59 patients (35.8%) to A2, 19 patients (11.5%) to B1, and 20 patients (12.1%) to B2 group.

The demographic characteristics and clinical variables of patients with and without PPMI were similar except glomerular filtration rate (GFR) and creatinine values and given in Table 1. Variables with a *p*-value < 0.1 in univariate analyses were considered for entry into the multivariate logistic regression model. Post-procedural variables were excluded from this selection process. Angiographic features of both patients with and without periprocedural MI are shown in the Table 2. The usage rates of beta blockers, calcium channel blockers, and RAS blockers in PPMI (+) and PPMI (−) patients were 20.3% vs. 17.6% (*p* = 0.812), 22.6% vs. 18.4% (*p* = 0.61), and 22.3% vs. 13.3% (*p* = 0.42), respectively.

**Table 1:** Demographic and clinical features of patients with and without PPMI.

Variables	All (*n* = 165)	No PPMI (*n* = 135)	PPMI (*n* = 30)	*p* value
Age (years, mean ± SD)	59 ± 9.4	59 ± 9.1	59 ± 10.8	0.975
Sex (male, %)	127 (77%)	104 (77%)	23 (77%)	0.965
DM (n, %)	60 (36.4%)	48 (37%)	12 (40%)	0.647
HT (n,%)	84 (51%)	69 (51%)	15 (50%)	0.912
CAD (n,%)	105 (64%)	90 (66.7%)	15 (50%)	0.067
FU (days, median-IQR)	368 (3–691)	368 (3–680)	367 (3–691)	0.266
Cr (mg/dl, mean ± SD)	0.9 ± 0.2	0.8 ± 0.1	0.9 ± 0.2	0.011*
GFR (ml/minute/m^2^, mean ± SD)	92.9 ± 18.2	94.0 ± 14.0	83.5 ± 17.0	0.031*
Hgb (gr/dl, mean ± SD)	13.4 ± 1.7	13.2 ± 1.6	13.1 ± 2	0.743
WBC (mcL, mean ± SD)	8.4 ± 2.1	8.3 ± 1.9	9.0 ± 3.0	0.536
HCT (mean ± SD)	38.8 ± 4.8	38 ± 4.2	38 ± 5.6	0.935
AST (IU/L, mean ± SD)	22.3 ± 16.1	20.5 ± 10.8	27.0 ± 25.0	0.355
Baseline hs-TnT (pg/ml)	0.024 ± 0.012	0.022 ± 0.010	0.036 ± 0.304	0.06
Post-PCI 12th hour hs-TnT (pg/ml)	0.356 ± 0.178	0.273 ± 0.140	0.850 ± 0.410	0.0001
Post-PCI 24th hour hs-TnT (pg/ml)	0.680 ± 0.304	0.585±	1.238 ± 0.265	0.0001
ALT(IU/L, mean ± SD)	22.1 ± 13.7	21.7 ± 15.1	22.9 ± 9.4	0.220
LVEF (%, mean ± SD)	56.8 ± 5.7	57.0 ± 5.8	56.5 ± 5.0	0.285
LA (mm, mean ± SD)	37.9 ± 4.4	37.9 ± 4.5	37.7 ± 4.3	0.916
RV (mm, mean ± SD)	23.5 ± 2.2	23.4 ± 2.3	23.4 ± 1.9	0.590

AST, Aspartate aminotransferase; ALT, Alanine transaminase; Cr, Creatinine; DM, Diabetes Mellitus; FU, Follow-up; GFR, Glomerular filtration rate; Hgb, Hemoglobin; hs-TnT, high sensitive troponin; HT, Hypertension; HCT, Hematocrit; LA, Left atrium; LVEF, Left ventricular ejection fraction; PPMI, Peri-procedural myocardial infarction; RV, Right ventricular diameter; WBC, White Blood Cell.

* Indicates variables with significant *p* < 0.05.

**Table 2:** Angiographic features of patients with and without periprocedural MI.

Variables	All (*n* = 165)	No PPMI (*n* = 135)	PPMI (*n* = 30)	*p* value
Lesion Type				
- Simple (A, B1) (*n*,%)	88 (53.3%)	78 (57.8%)	10 (33.3%)	0.015*
- Complex (B2, C) (*n*,%)**	77 (46.7%)	57 (42.2%)	20 (66.7%)	
Stent length (mm, mean ± SD)	22 ± 5.0	22 ± 4.0	22 ± 4.5	0.674
Stent diameter (mm, mean ± SD)	2.80 ± 0.25	2.8 ± 0.30	2.8 ± 0.30	0.879

PPMI, Peri-procedural myocardial infarction; SD, standard deviation.

* Indicates variables with significant. *p* < 0.05.

** Indicates lesion type classification according to ACC/AHA classification of coronary lesion.

The culprit lesion was on left anterior descending artery (LAD), right coronary artery (RCA) and circumflex artery (Cx) in 35%, 25%, and 22% of study population, respectively. The rest 18% patients had culprit lesion on side branch (diagonal, obtuse marginal, intermediary artery), left main coronary artery (LMCA) or by-pass graft. Intracoronary imaging (intracoronary ultrasound-IVUS) was used in just LMCA PCI procedures due to re-imbursement issue. Although the stent diameter and length were similar between 2 groups, patients with PPMI had more complex lesion (B2 and C). The relationship between the loading dose of rosuvastatin and periprocedural MI in statin naive patients and patients with already on statin is shown in Table 3. As seen from the Table 3, the incidence of PPMI is higher in statin-naive patients than statin-users (9/39, **23%** vs. 21/126, **17%**), but it was not statically significant (*p* > 0.05). The incidence of PPMI was **20.9%** in group **A1**, **11.9%** in group **A2**, **26.3%** in group **B1**, and **20%** in group **B2**. Most interestingly, the frequency of events in patients who received a loading dose of rosuvastatin, whether they were already using statins or statin-naive, was higher than in those who did not receive the loading dose. There was no preventive effect of rosuvastatin loading dose on periprocedural MI. There was no significant difference between the two groups (PPMI + vs. PPMI-) regarding dual antiplatelet treatment (DAPT) (100% vs. 100%, *p* = 1.0), beta-blocker use (80.6% vs. 77.8%, *p* = 0.812), calcium channel blocker (CCB) use (22.6% vs. 17.8%, *p* = 0.06), and renin-angiotensin system inhibitor (RAS) use (80.6% vs. 69.0%, *p* = 0.269).

**Table 3:** The relationship between rosuvastatin loading and periprocedural MI in statin naive patients and statin users.

Variables	No PPMI (*n* = 135)	PPMI (*n* = 30)	*p* value
Treatment Groups			
A1 (*n*, %)	53 (79.1%)	14 (20.9%)	0.436
A2 (*n*, %)	52 (88.1%)	7 (11.9%)	
B1 (*n*, %)	14 (73.7%)	5 (26.3%)	
B2 (*n*, %)	16 (80.0%)	4 (20.0%)	

A1: statin users with rosuvastatin loading, A2: statin users without rosuvastatin loading.

B1: statin naïve patients with rosuvastatin loading, B2: statin naïve patients without rosuvastatin loading.

PPMI, Peri-procedural myocardial infarction.

Table 4 presents the results of a logistic regression analysis examining the relationships between glomerular filtration rate (GFR) and lesion complexity with the incidence of peri-procedural myocardial infarction (PPMI). Patients with complex lesions have 3.401 times higher odds of experiencing periprocedural MI compared to those without complex lesions, and this result is statistically significant. For each unit increase in GFR, the odds of experiencing PPMI decrease by approximately 3%. This inverse relationship is statistically significant, suggesting that better renal function (higher GFR) is associated with a reduced risk of PPMI.

**Table 4:** GFR and lesion type relationships with PPMI.

Term	β estimates with standard errors	OR with 95% CI	*p* value
Constant $\left(\beta_{0}\right)$	0.656 ± 1.122	-	0.559
Complex Lesion type $\left(\beta_{1}\right)$	1.224 ± 0.445	3.40 (95% CI: 1.38–8.38)	0.006
GFR $\left(\beta_{2}\right)$	−0.031 ± 0.013	0.97 (95% CI: 0.95–0.99)	0.015

GFR, Glomerular filtration rate; OR, Odds ratio; PPMI, Peri-procedural myocardial infarction.

Table 4 underscores the importance of complex lesion types and renal function in predicting the risk of PPMI in patients undergoing PCI. It highlights that complex lesions significantly increase the risk, while better renal function (indicated by higher GFR) reduces the risk. Creatinine was eliminated from the stepwise procedure after GFR entered the model (due to the association between GFR and creatinine).

Just 2 patients (1.2%) experienced an MACCE during the follow-up period. One of them who had type C lesion was in group A2 and the event was TVR on day 391th. The other patient with type B1 lesion was in group A1 and was hospitalized due to ACS on the 40th day of follow-up.

## Discussion

The primary finding of our study highlights a notable discrepancy in the incidence of PPMI events between patients who were previously not using statins (23%) compared to those who were (17%) within a patient cohort diagnosed with stable CAD, but without CKD or HF, and undergoing PCI. Notably, during the follow-up period, only two MACCE were recorded. One event occurred in statin-prevalent patients who did not receive a loading dose of rosuvastatin, while the other event occurred in statin-naive patients who did receive a loading dose of rosuvastatin. Although, our study primarily aimed to evaluate the effect of high loading doses of rosuvastatin on PPMI incidence, Additionally, we conducted an exploratory analysis to identify characteristics associated with PPMI. It is important to note that this secondary analysis was not pre-defined and should be considered hypothesis-generating. We performed multiple comparisons, which introduces the risk of Type I error. While our findings suggest associations between certain characteristics and PPMI, these results should be interpreted with caution due to the potential for multiple comparison bias. Future studies with pre-defined hypotheses and appropriate adjustments for multiple comparisons are needed to confirm these findings. In our study, we focused on identifying baseline predictors of PPMI using a multivariate logistic regression model. Only pre-procedural variables with a *p*-value < 0.1 in univariate analyses were included in the model, ensuring that the predictors were relevant and available before the PCI procedure. This approach allowed us to identify independent predictors of PPMI while avoiding the inclusion of outcome variables, such as post-PCI hs-TnT levels.

When comparing our study population with others, the incidence of PPMI, occurring in approximately 15%–20% of patients undergoing PCI, underscores its significant clinical challenge due to its association with short- and long-term mortality (1). Zeitouni et al. demonstrated in a prospective observational study involving 1390 patients that a quarter of those undergoing elective PCI experienced periprocedural events, leading to longer hospitalizations and worse 30-day clinical outcomes (14). However, it is important to emphasize that our study population is composed of patients from a single-center university hospital in Istanbul, Turkey, and does not reflect the entire regional area of Turkey.

Unlike many other studies, our research adhered strictly to the “Fourth Universal Definition of Myocardial Infarction” guideline of the ESC, defining PPMI as a five-fold increase in troponin values (15–18). This adherence to standardized definitions is a major strength of our study. Briguori et al. similarly adopted this definition in a study involving 451 consecutive PCI patients, revealing PPMI rates of 23.5% in the statin group and 32% in the control group (19). In contrast, our study observed a lower PPMI incidence of 18.2%, with only 2 MACCE events occurring in patients without PPMI during the median 368-day follow-up period.

Numerous randomized studies have suggested a preventive effect of preprocedural statin therapy against PPMI, although further evidence is needed for conclusive confirmation (16, 18, 20–28). The mechanism behind this protective effect lies in the stabilization of plaques and inhibition of microembolism during PCI, particularly pronounced in acute coronary syndromes (ACS) characterized by increased inflammation and thrombotic plaque tendency (24). Rosuvastatin, recognized for its potency, has shown promise in reducing PPMI rates in various studies (23, 10).

However, the efficacy of single-dose statin therapy in preventing PPMI remains less studied in chronic statin users. Takano et al. revealed that high-dose statin therapy was more effective than low dose in preventing PPMI in statin-naive patients, while no difference was observed in chronic statin users (28). Similarly, Di Sciascio et al. found that high-dose atorvastatin was associated with lower PPMI rates in both stable and ACS patients (18).

In our study, regardless of statin use, high loading doses of rosuvastatin before PCI had no effect on PPMI incidence. Unadjusted analyses showed a clear association between high creatinine, low GFR, and lesion complexity with PPMI. However, it is important to note that the adjusted logistic regression model did not include creatinine, suggesting no discernible relationship between creatinine and PPMI after accounting for lesion type and GFR. This indicates that while creatinine appears to be related to PPMI in unadjusted analyses, this relationship does not hold when potential confounding factors are considered. Additionally, the majority of our patients receiving chronic statin therapy suggested an increased risk for ischemic heart diseases, potentially impacting the preventive effect of statin loading before the procedure. Veselka et al. similarly found that high-dose statin loading had no effect on PPMI incidence in statin-naive stable coronary artery patients (29).

We observed that lower GFR, and complex lesion types were clearly associated with an increased incidence of PPMI. These findings are consistent with existing literature, which suggests that renal function and lesion complexity are significant predictors of periprocedural complications (30, 31). For instance, studies have shown that impaired renal function, as indicated by low GFR, is a risk factor for adverse cardiovascular events, including PPMI, due to its association with atherosclerosis and vascular calcification (30, 31). Similarly, complex coronary lesions have been linked to higher rates of procedural complications and adverse outcomes (32, 33). Understanding these associations is crucial for clinical practice, as it highlights the need for careful preprocedural assessment and optimization of patients with these risk factors. This may include more aggressive medical management, close monitoring, and potentially alternative interventional strategies to mitigate the risk of PPMI in high-risk patients.

However, our results are not consistent with some studies that have demonstrated a protective effect of high-dose statins against PPMI. For example, a meta-analysis by de Liyis et al. (2024) showed a notable decrease in MACE with high-dose statin loading before PCI (34). Their pooled results, encompassing 6,207 patients, indicated a significant reduction in MACE at three months post-PCI (OR 0.50, 95% CI 0.35–0.71, *p* = 0.0001). This discrepancy may be attributed to differences in study populations, the acute coronary syndrome (ACS) focus of the meta-analysis, and variations in follow-up durations. Our study’s focus on stable CAD and a shorter follow-up period may partly explain the lack of observed benefit. These findings suggest that while high-dose statins are beneficial in ACS settings, their role in stable CAD requires further exploration with larger cohorts and extended follow-up. Additionally, Veselka et al. found that high-dose statin loading had no effect on PPMI incidence in statin-naive stable coronary artery patients (29). Our findings are consistent with this study, highlighting the complexity of statin effects in different clinical settings.

In summary, while our study contributes to the understanding of statin loading in PCI, it also underscores the variability in outcomes across different patient populations and study designs. Future research should focus on identifying patient-specific factors that influence the efficacy of statin therapy and on designing studies with larger cohorts and longer follow-up periods to provide more definitive conclusions.

Our study has several limitations that warrant acknowledgment. Firstly, the lack of blinding in our study design may introduce potential biases into our findings, as both patients and clinicians were aware of the treatment assignment. This lack of blinding could have influenced the reporting of outcomes and introduced an element of bias into our results. Additionally, our study may have lacked adequate statistical power to comprehensively assess the primary outcome of interest, namely the occurrence of PPMI. While our findings suggest a trend towards a higher incidence of PPMI among statin-naïve patients compared to statin users, the limited sample size and duration of follow-up may have hindered our ability to detect statistically significant differences. Moreover, our study was not powered to assess the occurrence of MACCE, which represents an important clinical outcome. Specifically, patient enrollment and study duration were impacted by the COVID-19 pandemic and other logistical challenges. Due to the COVID-19 pandemic, we had to keep the number of patients and FU duration shorter than we expected. Therefore, caution should be exercised in interpreting our results, particularly with regard to the impact of statin loading doses on MACCE. To address these limitations, further studies with larger cohorts and longer follow-up periods are warranted to provide more robust evidence regarding the effects of statin loading doses on both PPMI and MACCE. Additionally, comparisons between statin users and statin-naive patients were subject to potential confounding factors. Despite adjusting for some confounding variables, residual confounding may still be present, which could affect the robustness of our conclusions. Our exploratory analysis aimed to identify characteristics associated with PPMI, which was not pre-defined and involved multiple comparisons. This increases the risk of Type I error, and the findings should be considered hypothesis-generating. Future research should include pre-defined hypotheses and appropriate adjustments for multiple comparisons. Finally the study population are included from a tertiary university hospital in Istanbul Turkey, and does not reflect the entire Turkish population. Despite these limitations, our study adhered to the guidelines set forth by the ESC for defining PPMI, which enhances the validity and reliability of our findings. This adherence underscores the importance of considering various clinical, ethnic, and study design factors in the interpretation of our results and highlights the need for future research to provide more definitive insights into this complex clinical scenario.

## Conclusion

In this randomized single-center study, the administration of pre-procedural high-dose rosuvastatin (40 mg) did not demonstrate efficacy in preventing PPMI, regardless of chronic statin use. Our findings shed light on the nuanced interplay between pharmacotherapy and procedural outcomes, underscoring the need for a multifaceted approach in managing patients undergoing PCI.

Despite the lack of preventative efficacy observed with rosuvastatin loading, our study uncovered valuable insights into the characteristics associated with PPMI. Specifically, we identified associations between PPMI occurrence and high levels of creatinine, low GFR, and the complexity of coronary lesions. These findings highlight the multifactorial nature of PPMI and underscore the importance of considering patient-specific factors in risk stratification and management strategies.

While our study contributes to the growing body of evidence regarding the use of statins in PCI, further research is warranted to elucidate the optimal pharmacotherapeutic approaches for mitigating PPMI and improving patient outcomes. Future studies incorporating larger cohorts and longer follow-up periods may provide additional clarity on the role of statins in this context, ultimately informing clinical practice and optimizing patient care.

## Data Availability

The raw data supporting the conclusions of this article will be made available by the authors, without undue reservation.
